# Supplementary material for: Shrimp genome sequence contains independent clusters of ancient and current Endogenous Viral Elements (EVE) of the parvovirus IHHNV
Source: BMC Genomics. 2022 Aug 6;23:565. doi: 10.1186/s12864-022-08802-3 (PMC9357335; doi:10.1186/s12864-022-08802-3)
Supplement: Supplementary file 1 — Additional file 1: Supplementary Figure S1. Alignment of the 561 bp repetitive sequences flanking the non-infectious IHHNV-EVE Cluster 1 located in PC35. The sequences are aligned in the same direction and share 98% identity (547/561 bp with 9/561 nucleotide gaps). Supplementary Figure S2. Alignment of the two 519 bp repetitive sequences flanking IHHNV-EVE Cluster 2 located on PC35. The sequences are aligned in the same direction and share 77% identity (400/519 bp with 66/519 nucleotide gaps). Supplementary Figure S3. Alignment of the direct repetitive sequences flanking the infectious IHHNV-EVE Cluster 3 located on PC7. A total 1758 bp were aligned and share 98% identity (1722/1758 bp with 21/1758 nucleotide gaps). Supplementary Figure S4. Multiple sequence alignment of potential 309 bp targets for the OIE- recommend IHHNV detection method from three repeats in IHHNV-EVE observed in PC7 (R1-R3). The 309F/R primer positions are indicated by red rectangles. The primer sequences and potential amplicon sizes match 100% to those expected for infectious IHHNV. Supplementary Figure S5. Multiple nucleotide sequences alignment of PCR amplicons of R1 fragment in Fig. 4. Both forward and reverse sequence read directions were analyzed and the sequence similarity was 99% identity when aligned to original PC7 sequence portion. The nucleotide portion in rectangular represent anti-sense direction of IHHNV-EVE corresponding to the PC7 sequence assembly. [file 12864_2022_8802_MOESM1_ESM.docx]

**Supplementary information**


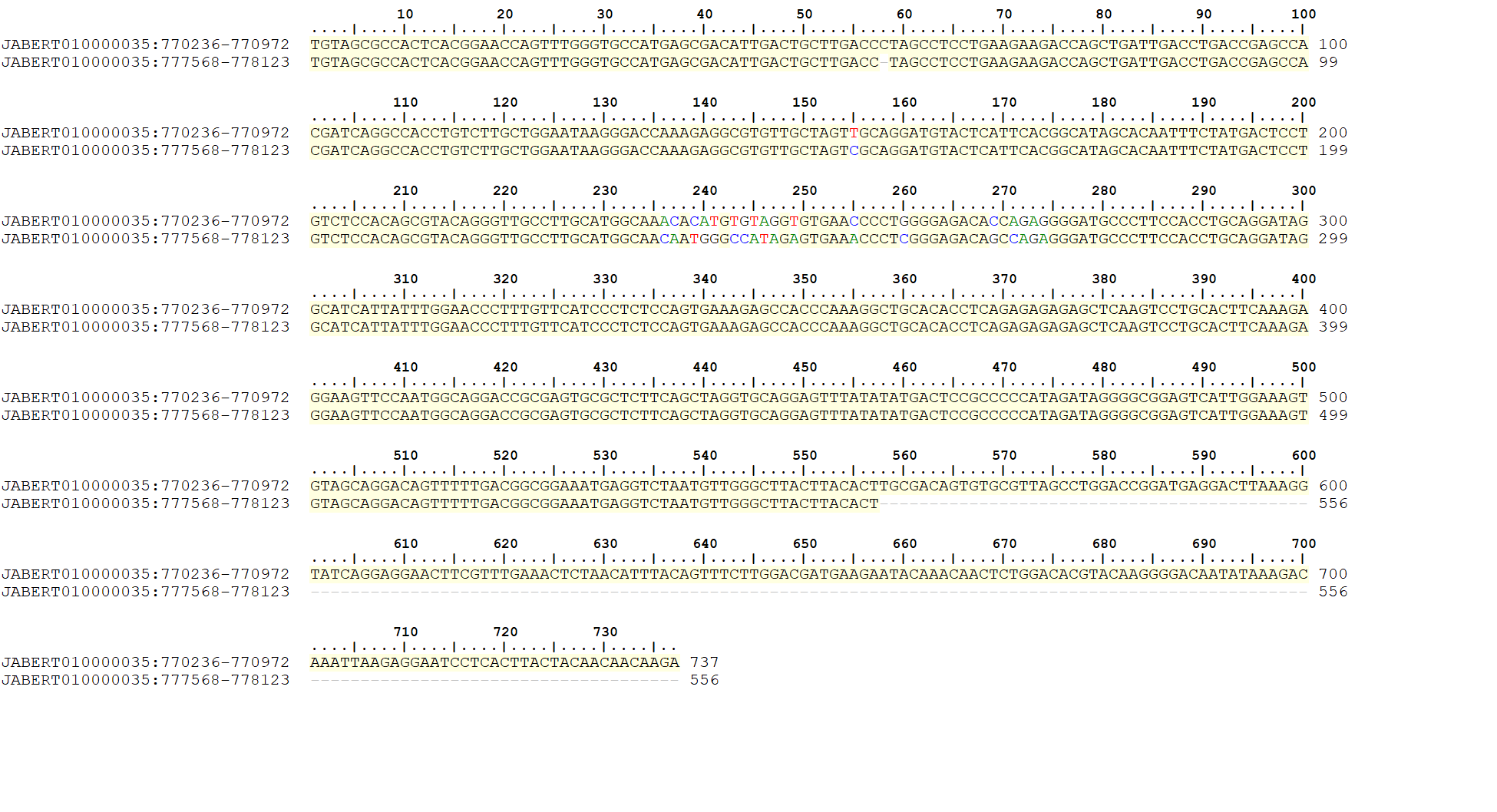


**Supplementary Figure S1.** Alignment of the 561 bp repetitive sequences flanking the non-infectious IHHNV-EVE Cluster 1 located in PC35. The sequences are aligned in the same direction and share 98% identity (547/561 bp with 9/561 nucleotide gaps).


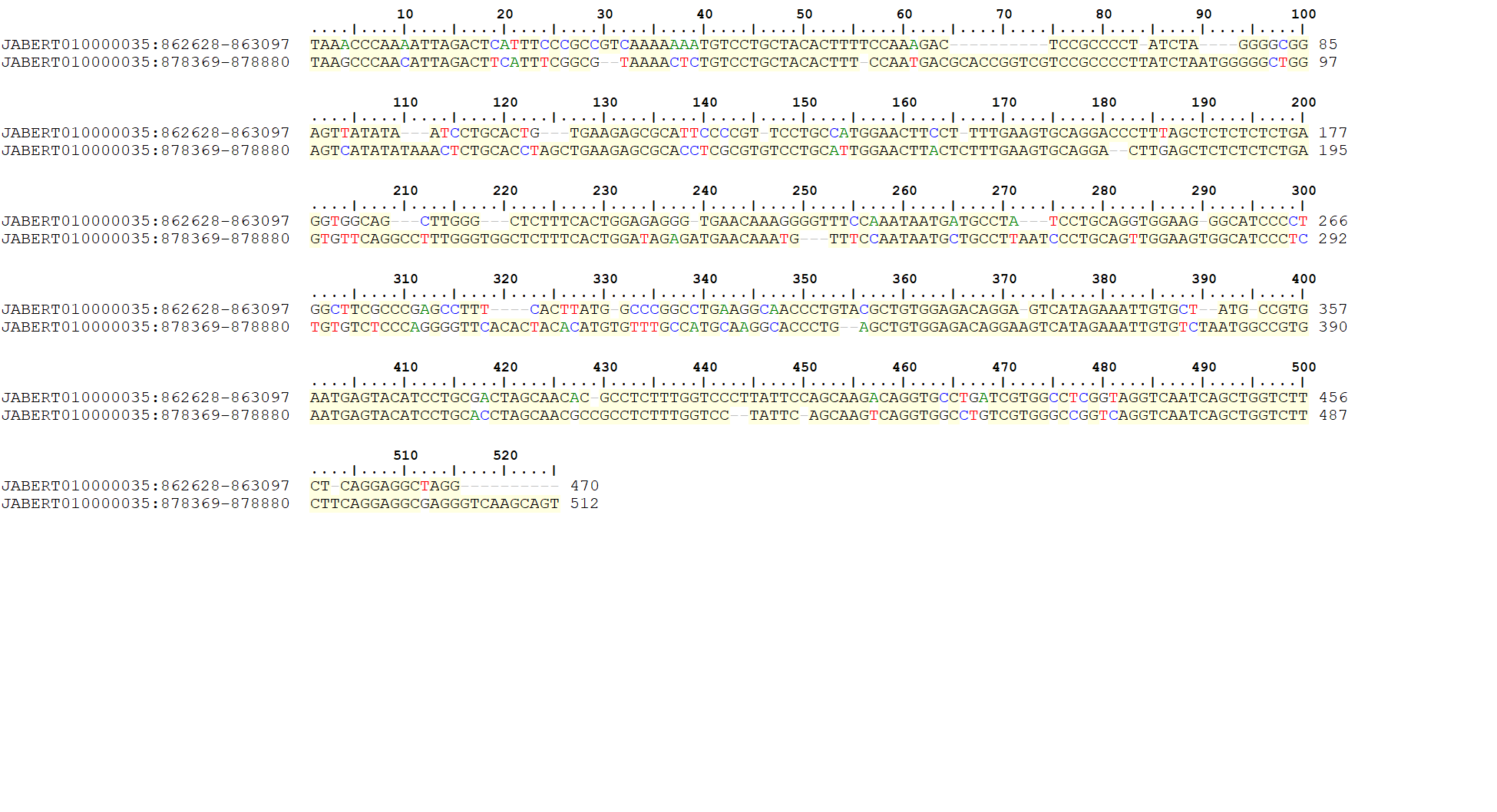


**Supplementary Figure S2.** Alignment of the two 519 bp repetitive sequences flanking IHHNV-EVE Cluster 2 located on PC35. The sequences are aligned in the same direction and share 77% identity (400/519 bp with 66/519 nucleotide gaps).


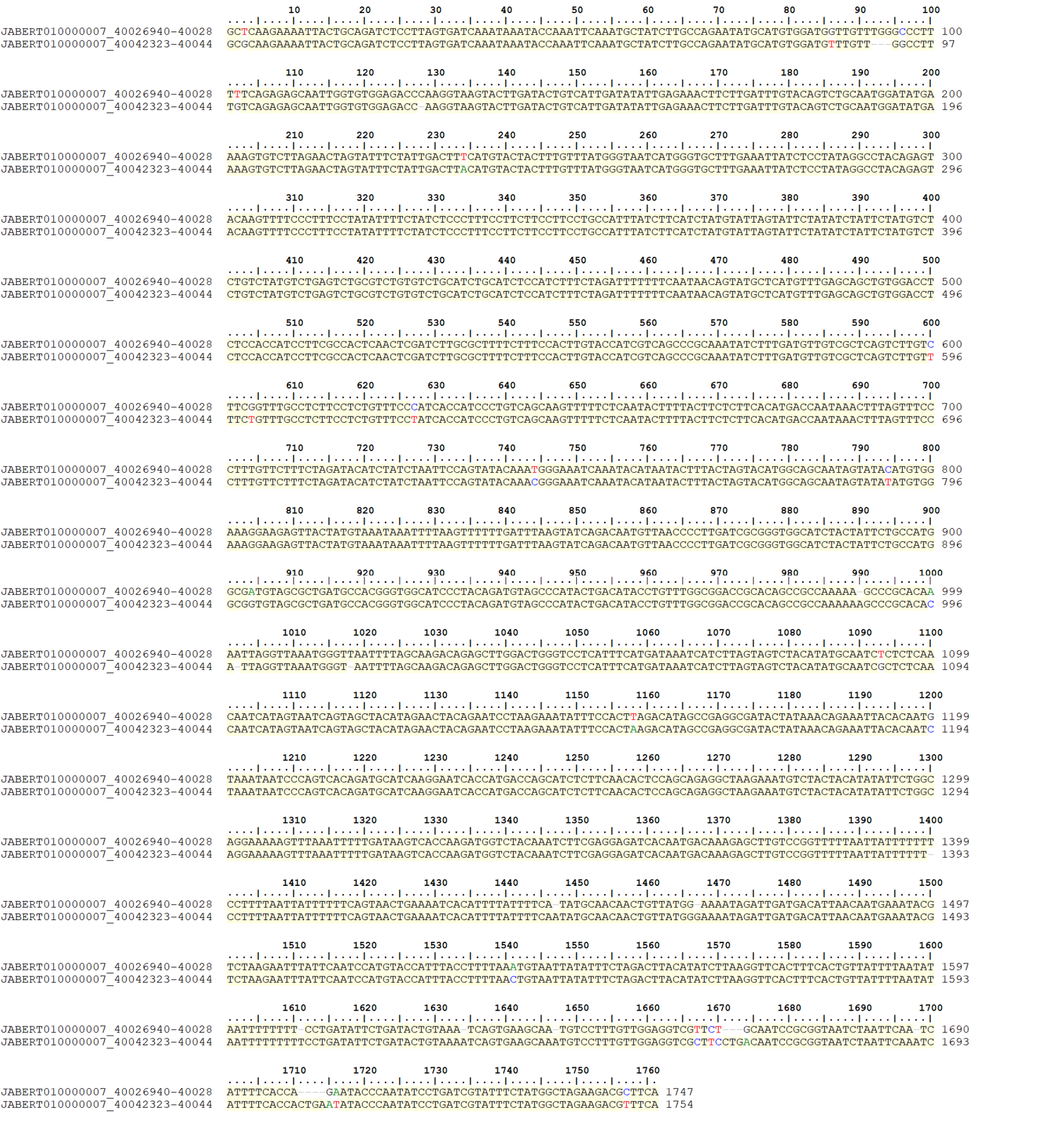


**Supplementary Figure S3.** Alignment of the direct repetitive sequences flanking the infectious IHHNV-EVE Cluster 3 located on PC7. A total 1,758 bp were aligned and share 98% identity (1722/1758 bp with 21/1758 nucleotide gaps).

**
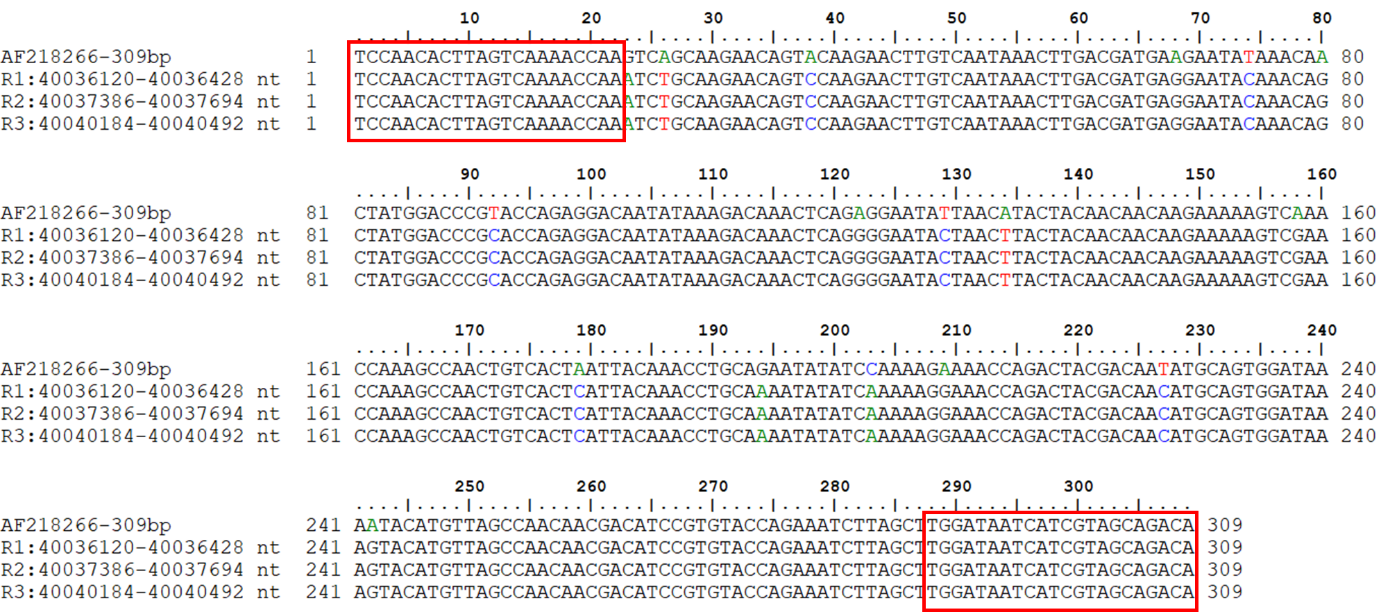
**

**Supplementary Figure S4.** Multiple sequence alignment of potential 309 bp targets for the OIE- recommend IHHNV detection method from three repeats in IHHNV-EVE observed in PC7 (R1-R3). The 309F/R primer positions are indicated by red rectangles. The primer sequences and potential amplicon sizes match 100% to those expected for infectious IHHNV.


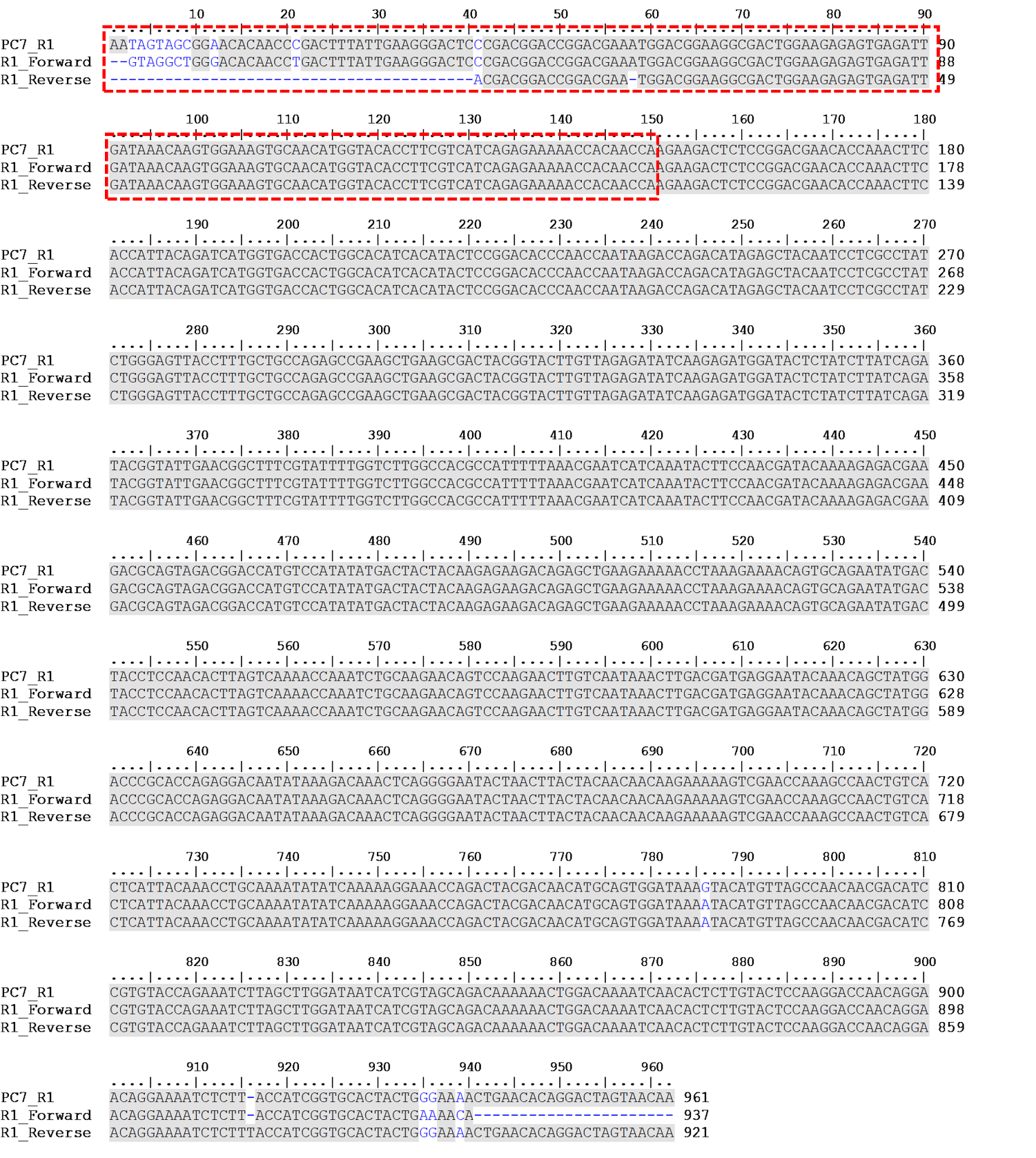


**Supplementary Figure S5.** Multiple nucleotide sequences alignment of PCR amplicons of R1 fragment in Fig. 4. Both forward and reverse sequence read directions were analyzed and the sequence similarity was 99% identity when aligned to original PC7 sequence portion. The nucleotide portion in rectangular represent anti-sense direction of IHHNV-EVE corresponding to the PC7 sequence assembly.
